# BaMBa: towards the integrated management of Brazilian marine environmental data

**DOI:** 10.1093/database/bav088

**Published:** 2015-10-10

**Authors:** Pedro Milet Meirelles, Luiz M. R. Gadelha, Ronaldo Bastos Francini-Filho, Rodrigo Leão de Moura, Gilberto Menezes Amado-Filho, Alex Cardoso Bastos, Rodolfo Pinheiro da Rocha Paranhos, Carlos Eduardo Rezende, Jean Swings, Eduardo Siegle, Nils Edvin Asp Neto, Sigrid Neumann Leitão, Ricardo Coutinho, Marta Mattoso, Paulo S. Salomon, Rogério A.B. Valle, Renato Crespo Pereira, Ricardo Henrique Kruger, Cristiane Thompson, Fabiano L. Thompson

**Affiliations:** ^1^Institute of Biology, Federal University of Rio de Janeiro (UFRJ), Av. Carlos Chagas Filho 373 Sala A1-050, Bloco A do CCS Cidade Universitária, 21941-902 - Rio de Janeiro, RJ, Brazil,; ^2^Federal University of Rio de Janeiro (UFRJ) / COPPE, SAGE, Rua Moniz Aragão 360, Bloco 2, Ilha do Fundão, 21945-972 - Rio de Janeiro, RJ, Brazil,; ^3^National Laboratory for Scientific Computing (LNCC), Av. Getúlio Vargas 333, Quitandinha, 25651-075 - Petropolis, RJ, Brazil,; ^4^Department of Environment and Engineering, Federal University of Paraíba, Rio Tinto, Brazil (UFPB), Rua da Mangueira, s/n - Campus IV (Litoral Norte), Centro, 58297-000 - Rio Tinto, PB, Brazil,; ^5^Rio de Janeiro Botanical Garden Research Institute (IP-JBRJ), Rua Pacheco Leão 915, Horto, 22460-030 - Rio de Janeiro, RJ, Brazil,; ^6^Department of Oceanography and Ecology, Federal University of Espírito Santo (UFES), Av. Fernando Ferrari, 514, Goiabeiras, 29090-600 - Vitória, ES Brazil,; ^7^Environmental Sciences Laboratory (LCA), Northern Rio de Janeiro State University Darcy Ribeiro (UENF), Avenida Alberto Lamego 2000, Parque Califórnia, 28013-602 - Campos dos Goytacazes, RJ, Brazil,; ^8^Oceanographic Institute, University of São Paulo (IO-USP), Praça do Oceanográfico, 191, Cidade Universitária, 05508-120 - Sao Paulo, SP, Brazil,; ^9^Institute of Coastal Studies, Federal University of Para (UFPA), Alameda Leandro Ribeiro, s/n. - Bairro Aldeia, UFPA/Campus Universitário de Bragança Aldeia, 68600-000 - Braganca, PA, Brasil,; ^10^Department of Oceanography, Federal University of Pernambuco (UFPE), Av Arquitetura, S/N, Cidade Universitaria, 50670-901 - Recife, PE, Brazil,; ^11^Division of Marine Biotechnology, Marine Studies Institute Admiral Paulo Moreira, Rua Kioto 253, Praia dos Anjos, 28930-000 - Arraial do Cabo, RJ, Brazil,; ^12^PESC/COPPE - Federal University of Rio de Janeiro, Centro de Tecnologia, Bloco H, sala 319, Ilha do Fundão, 21941972 - Rio de Janeiro, RJ, Brazil,; ^13^Departament of Marine Biology, Federal Fluminense University (UFF), Morro do Valonguinho s/n, Centro, 24001-970 - Niteroi, RJ, Brazil, and; ^14^Laboratory of Enzymology, Department of cellular Biology, Institute of Biology, University of Brasília (UnB), Asa Norte 70910-900 - Brasília, DF – Brazil

## Abstract

A new open access database, Brazilian Marine Biodiversity (BaMBa) (https://marinebiodiversity.lncc.br), was developed in order to maintain large datasets from the Brazilian marine environment. Essentially, any environmental information can be added to BaMBa. Certified datasets obtained from integrated holistic studies, comprising physical–chemical parameters, -omics, microbiology, benthic and fish surveys can be deposited in the new database, enabling scientific, industrial and governmental policies and actions to be undertaken on marine resources. There is a significant number of databases, however BaMBa is the only integrated database resource both supported by a government initiative and exclusive for marine data. BaMBa is linked to the Information System on Brazilian Biodiversity (SiBBr, http://www.sibbr.gov.br/) and will offer opportunities for improved governance of marine resources and scientists’ integration.

Database URL: http://marinebiodiversity.lncc.br

## Introduction

Marine sciences are increasingly supported by advanced computational infrastructures both in terms of processing power (1) and data management and storage capabilities (2). The availability of data on biodiversity and ecology is growing at a fast rate (3, 4) through global-scale data infrastructures such as Data Observation Network for Earth (DataONE) (5), the Global Biodiversity Information Facility (GBIF) (6) and the Ocean Biogeographic Information System (OBIS) (7). However, the complexity and range of environmental research data makes data management a difficult task (8). For instance, integrated reef systems studies require concomitant measurement of water and sediment chemistry, microbiology, -omics [i.e. (meta)genomics, (meta)transcriptomics and (meta)proteomics], benthic and fish surveys (9, 10). Despite a significant existing number of databases, an integrative database exclusive for marine environments does not exist yet. Current databases such as IMG (11), the Genomes OnLine Database (GOLD) (12) and the Metagenomic Rapid Annotation using Subsystem Technology (MG-RAST) (13) allow -omics and metadata deposition, but lack important data from other compartments of the system, for the sustainable maintenance of the marine realm (e.g. macro- organismal and plankton data). IMG is a microbial genome and metagenome data management system that implements a data warehouse to store and analyse metagenomic sequences both functionally and taxonomically. MG-RAST is a large repository with over than 150 000 datasets and multiple search mechanisms that provides a metagenomics-processing pipeline through a web-based interface, also allowing for taxonomic and functional classification of sequenced organisms. The Brazilian Marine Biodiversity Database (BaMBa) was developed to allow for the publication and exploration of marine biodiversity data, including biotic, abiotic, and -omic sample analyses and species distributions. It provides dataset repositories for publishing data in standardized formats and an integrated database that harvest these datasets to provide an integrated view of marine biodiversity. Techniques and data models developed in previous works involving ecological data management, such as the Brazilian Information System on Antarctic Environmental Research (14) and the Guanabara Bay Long Term Ecological Research Database (15), were utilized in the development of BaMBa. These previous systems did not consider, however, the management of -omics data, which is now contemplated by BaMBa.

BaMBa was built and is being extended within a larger initiative of the Brazilian National Research Network on Marine Biotechnology (BiotecMar). The system is also connected to the Brazilian Biodiversity Information System (SiBBr) (http://www.sibbr.gov.br), an initiative of the Brazilian Ministry of Sciences (MCTI) in partnership with National Laboratory for Scientific Computing (LNCC), where long term and secure storage of data is guaranteed. Therefore, BaMBa is going to be useful in a national scientific, environmental, economic and governmental context. The tool will enable scientists working on the Economic Exclusive Zone (EEZ, the nearly 4.5 Million km^2^ surrounding the Brazilian coast) to deposit and analyse their data in an integrated manner (17). BaMBa will unite data providing information on dominant benthic habitats in different locations of the EEZ. Integrated information directly available to stakeholders provides guidance for environment usage governance. Regulation and optimization of marine resources exploitation (i.e fishing, mineral, oil and gas extraction) are also facilitated by the rapid use of open access available data. For instance, recent studies have demonstrated the ecological and economic relevance of the rhodolith beds in Abrolhos Bank and in the Vitória-Trindade Chain (VTC) (18–20).

It was our aim was to develop a new integrated database, the Brazilian Marine Biodiversity Database (BaMBa), which allows integrated views of different data types concerning Brazilian marine environment, and is a potential tool to be used for improving governance of marine resources. BaMBa is focused on carefully curated and secured marine datasets.

## Methods

**Figure 1.**
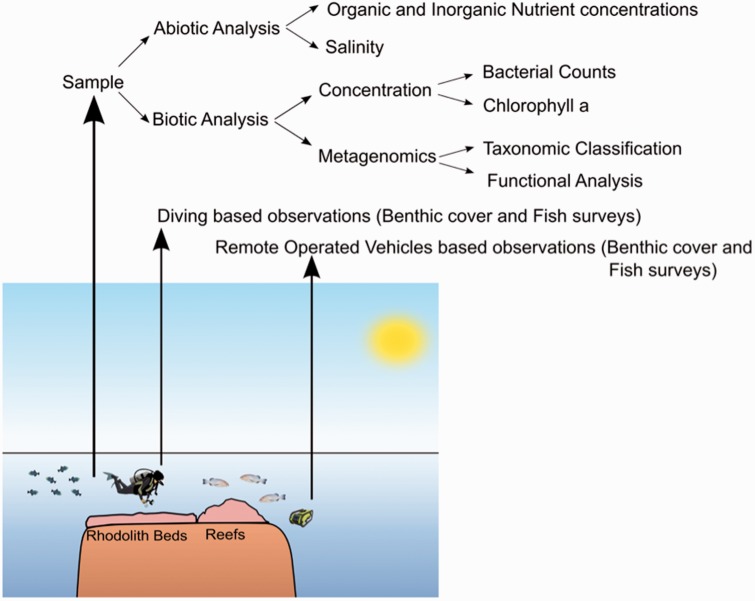
Research routine example of a multidisciplinary research group. Divers collect samples during scientific expeditions. These samples are analysed both for biotic and abiotic measurements. Divers and remote operating vehicles (ROVs) record observations (notes, photo or video).

BaMBa supports managing data obtained or derived from marine surveys such as the BiotecMar research group and others in an integrated manner. Sequences are extracted through high-throughput sequencing using water or material samples (Figure 1). These are processed by services, such as MG-RAST and Find Organisms by Composition USage (FOCUS) (21), for functional analysis and taxonomic classification. The samples are also analysed in a laboratory for biotic measurements, such as bacterial counts and chlorophyll concentrations, and for abiotic measurements, such as organic and inorganic nutrient concentrations and also for elemental and isotopic composition (e.g. δ^13^C, δ^15^N, δ^18^O and others) and several biomarkers (e.g. lipids, amino acids, lignin phenols, hydrocarbon, others). Isolation of microorganisms (prokaryotes and protists e.g. microalgae) is conducted from field samples of water or holobionts using traditional agar-plating techniques and cutting edge fluorescence-activated cell sorting. The obtained culture collections of microbial strains are kept either cryopreserved or by means of sequential transfers. Additionally, photos and videos generated during diving and by remotely operated vehicles are used for benthic cover and fish surveys (Figure 1). Integrating this data involves providing a unified view of data that originates from different sources (22), which is the case of BaMBa, where data involves -omics sequences, environmental measurements, and biodiversity monitoring. At the same time, the inclusion of physical parameters from the environment, such as temperature, salinity, circulation, waves, sediment properties, can be added in order to define the boundary conditions for surveyed organisms and processes.

**Figure 2.**
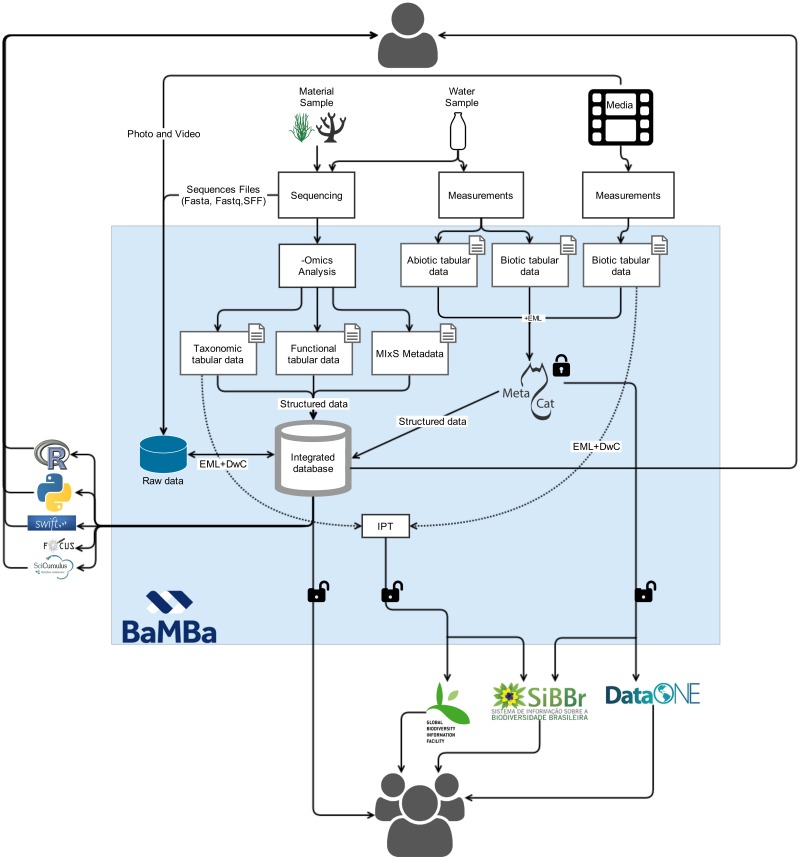
BaMBa system architecture. Media (photos and videos) and spreadsheets are uploaded into Marine Biodiversity Metacat system, which is recognized via EML to the database. (1) Users upload metadata and data (e.g. spreadsheets, FASTA files, compressed files and digital media files) using the BaMBa web interface or Morpho application; (2) the metadata (in EML format) and data uploaded by users is stored in BaMBa PostgreSQL relational database; (3) users can restrict data access or; (4) make it public. Once data is public BaMBa database automatically mirror it on other servers (5). Users can use tools like Python, R and FOCUS (21, 53, 58) to analyse and visualize deposited data.

**Figure 3.**
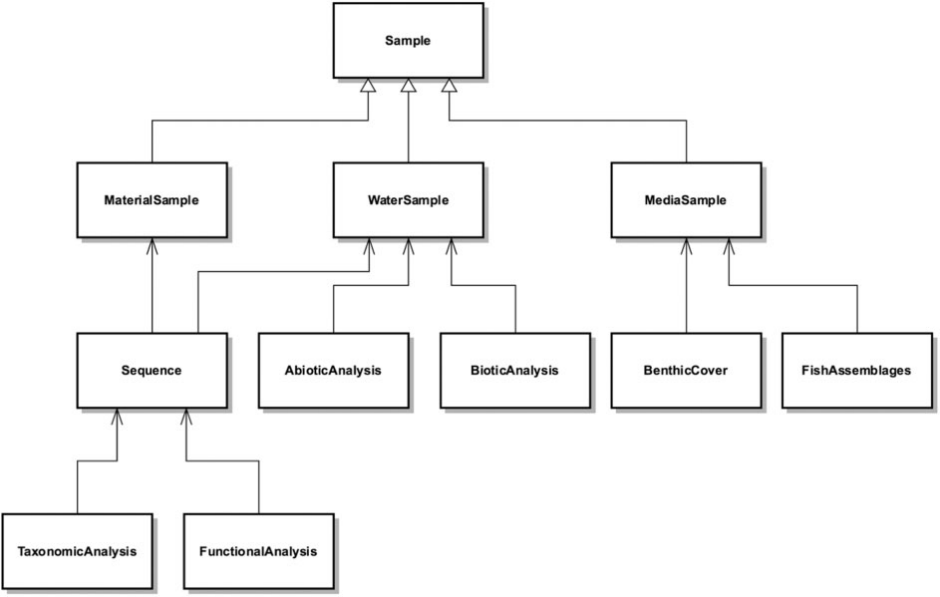
BaMBa schema.

The main concept behind the architectural design of BaMBa was to leverage existing data publishing and metadata standards for each of the content types managed in the system in order to facilitate data integration. As illustrated in Figure 2, the architecture of BaMBa is composed of the following main components:

- **Ecological data repository**. The Ecological Metadata Language (EML) was used in order to allow for the contextual description of tabular ecological datasets, including their taxonomic, temporal, and geographic coverage, and project and methodology description (23). Additionally, EML allows for describing tabular datasets at the column level, by describing, for instance, the content and unit of measurement. Metacat was used to create data and metadata repositories, which support EML, as the publishing tool for tabular ecological datasets (24). We chose Metacat application because: (i) it is an open source web application; (ii) the Web interface facilitates the input and retrieval of data; (iii) it stored datasets query and visualization of geographic cover thorough mapping functionality; (iv) datasets can be stored safely on multiple datasets using Metacat's replication feature; (v) easy customization capability; (vi) automation of retrieving and storing EML documents from other sites and (vii) built in logging system that allows curation and tracking for document insertions, deletions and reads. The new database works on a Linux web server located in National Laboratory for Scientific Computing (LNCC), connected to a PostgreSQL database. Data are stored in Metacat using an Extensible Markup Language (XML) format and EML standards. Any type of data can be uploaded to this repository by the users, using an easy web interface or Morpho application (25). EML allows data publishers to describe datasets instead of having to map attributes to a fixed database schema (26). The use of Metacat allows for further dissemination of the datasets to the DataONE (5) network and the *Ecological Data Portal* of the Brazilian Biodiversity Information System (SiBBr) (27). BaMBa’s ecological data repository can be accessed at https://marinebiodiversity.lncc.br/metacatui.
- **Species occurrences repository**. An installation of the GBIF Integrated Publishing Toolkit (IPT) (28), hosted at SiBBr, is used to share species occurrence data. The datasets follow the Darwin Core standard for tabular data on species occurrences and the metadata follows the EML standard. These datasets are harvested by GBIF, SiBBr and BaMBa. BaMBa’s species occurrences repository can be accessed at http://www.gbif.org/publisher/6b6f5206-bbbc-4079-8685-ce5b664eaaf3
- **An integrated database for marine biodiversity.** A conceptual data model, illustrated in Figure 3, given by data entities that capture the breadth of research activities executed by BiotecMar. This database is currently implemented in PostgreSQL. The *Sample* entity contains attributes that are common to any sample, such as the locality (i.e. latitude and longitude), depth where and the date when it was collected. *MaterialSample*, *WaterSample* and *MediaSample* are specializations of the *Sample* entity and contain attributes that are specific to them, such as equipment used for recording a video in a *MediaSample*. The *Sequence* entity may be associated to a *MaterialSample* or a *WaterSample* and contains attributes defined in the MIxS standard, such as the sequencing method used. The sequences are analysed and produce tabular data on their taxonomic classification, which are attributes of the *TaxonomicAnalysis* entity, and on their functional classification, which are attributes of the *FunctionalAnalysis* entity. The *AbioticAnalysis* and *BioticAnalysis* entities are both associated to a *WaterSample*. They contain attributes such as salinity, hydrodynamic and meteorological parameters (e.g. current and wind speed and direction, rain) and organic and inorganic nutrient concentrations, for *AbioticAnalysis*, and bacterial and chlorophyll counts, for *BioticAnalysis*. Finally, the *BenthicCover* and *FishAssemblages*, which are associated to a *MediaSample*, contain attributes that describe population abundance for benthic and fish species respectively. This data model covers the current variety of data that is of interest to the research activities of in Marine Sciences, and will most likely need to evolve to meet new data content requirements. BaMBa’s integrated database for marine biodiversity can be accessed at https://marinebiodiversity.lncc.br/bamba/explore/

The data management policy adopted by BaMBa provides many options for data publication based on licenses from Creative Commons. Users also have the option of keeping their datasets private for some time, making only the metadata available initially. Other users can always contact the owners of a dataset to eventually obtain it directly from them.

## Results and Discussion

### Data publication workflow

Publishing data in BaMBa requires having an account in our private cloud file hosting service based on OwnCloud software (29) available at https://marinebiodiversity.lncc.br/files. Registered users can upload their data, such as spreadsheet files and photos, to this service. The only requisite to deposit data in BaMBa is to be from the marine environment. This data can be divided basically in two sets: (i) *Legacy datasets* produced by research expeditions prior to the adoption of the current data management practices do not follow any particular standard and, therefore, the formats used are very heterogeneous. (ii) *Standardized datasets*, are formatted using well- established data standards, such as MIxS (30), EML and Darwin Core, and produced using provided tabular data templates. Legacy datasets are published in Metacat, which requires an effort to document them using EML. Standardized datasets are composed of: species occurrences’ (using EML and Darwin Core, are published in IPT), biotic and abiotic analyses (using EML and spreadsheets filled using standard templates, are published in Metacat), functional and taxonomical analyses of metagenomes (using MIxS, are produced by MG-RAST). Both MIxS and EML are leveraged when mapping metagenomic analyses and ecological datasets to BaMBa's integrated database. Both standards overlap, for instance, in the description of research projects and methodologies. However they differ in attributes specific to their respective area. EML, for instance, allows for describing column-level attributes in ecological datasets. MIxS allows for describing attributes specific to genomics, such as the sequencing technology and methodology (i.e. whole genome shotgun sequencing, metatranscriptome for cDNA shotgun sequencing, or amplicon sequencing). A *harvesting* process is executed to retrieve the standardized datasets, which consist of file packages, from IPT and Metacat and to extract and import their content into the integrated database for marine biodiversity. This database can be explored through a web interface that allows for various queries to be executed that will return tabular views of the data. This database can be explored through a web interface that allows for various queries to be executed. In the Metacat instance of BaMBa there is a powerful search engine tool that allows users to search for a project, key words, species, and geographic location. Users can browse data and metadata in a web interface map, facilitating data sharing and visualization.

### Data curation and quality control

Data quality is a challenging issue in biodiversity data management (31). BaMBa implements both manual and automated data quality checks. The BaMBA curation team, checks the geographic coordinates of datasets prior to deposition, to certify that they belong to marine environments. Automated data curation practices in BaMBa include basic data quality checks, for instance, on the taxonomic names against reference checklists such as WoRMS (32) and the Catalogue of Life (33) on the location species occurrences against known species ranges. If some inconsistency or discrepancy is present, users will be notified and they can modify the data prior to deposition. If users choose to proceed the deposition, inconsistent or discrepant data is going to be marked in BaMBa as such.

### Data input and analysis

In principle any kind of file can be deposited in BaMBa. Four independent studies evaluated the usefulness of BaMBa. First, the fish surveys from the VTC dataset comprised mainly fish biodiversity data (16). This study was the largest check-list of reef fish for VTC. Second, a detailed morphological and phylogenetic data survey of campanulariids (Campanulariidae, Hydrozoa) (34). Third, the first microbial diversity study at VTC, including seamounts and island (17). This was the first comprehensive analysis of VTC, including metagenomic, water quality and benthic datasets. Four, a study about the microbial and viral dynamics of a costal Downwelling-Upwelling Transition (35). Although BaMBa database does not provide analytical tools, R package and Morpho, Kepler, and Swift can be applied to the outputs of the database. Users can use BaMBa metadata input model as an assistant to experimental design, making sure that data will be collected completely in an integrative way. BaMBa is connected to SiBBr, DataONE and GBIF, allowing rapid retrieval of information from any location in the Globe.

### BaMBa and other international biodiversity databases

BaMBa is the only integrated database resource both supported by a government initiative and exclusive for marine data (Table 1). The Biological Information System for Marine Life (BISMaL) (36), is an exclusive marine biodiversity database supported by a Japanese government initiative. It has a similar scope in terms of species occurrence records as BaMBa, however this repository does not embrace -omics and ecological data, as well environmental context of the samples. BISMaL works as the system of the Japan Regional OBIS Node (J-RON). OBIS is restricted to marine species biogeographic data, whereas WoRMS is restricted to marine species occurrence (32). Although very broad and embracing different types of environments and samples, MG-RAST is a powerful database and tool for analysing metagenomic data. The scopes of MG-RAST and BaMBa are different. BaMBa does not perform metagenome analysis. Instead, it consumes such data from tabular datasets produced by MG-RAST or any annotation system. There are some very useful databases for molecular genetic studies of natural marine populations (e.g. PhytoREF, GeoSymbio, *Littorina* Sequence Database, EvolMarkers) (37–40). However, these databases are focused on specific organisms or do not provide enough metadata. Broader databases like DataONE and The Knowledge Network for Biocomplexity (KNB) have powerful search engines (e.g. keywords, location, data attributes, publication date and taxon) and geographic browser but are not exclusive for marine data.

**Table 1.** MarineBiodiversity database is a stable, long term, secure and exclusive to marine-related data that harbors a great diversity of information and data types^a^

Feature	Marine Biodiversity	GIBF	ESA Data Registry	Dryad	The Knowledge Network for Biocomplexity	Tree BASE	WoRMS	OBIS	MG-RAST	PhytoREF	GeoSymbio	*Littorina* Sequence Database (LSD)	BISMaL
URL	http://marine biodiversity.lncc.br	http://www.gbif.org	http://data.esa.org	http://datadryad.org	http://knb.ecoinformatics.org	http://www.treebase.org	http://www.marinespecies.org	http://www.iobis.org	http://metagenomics.anl.gov	http://phytoref.org	https://sites.google.com/site/geosymbio/	http://mbio-serv2.mbioekol.lu.se/Littorina1/	http://www.godac.jamstec.go.jp/bismal/e/index.html
Funder	Brazilian Ministry of Science, Technology and Innovation (MCTI)	Funding agencies from each contributing country (33 countries)	Ecological Society of America (ESA)	NSF/The National Evolutionary Synthesis Center	NSF	NSF	Vlaams Instituut voor de Zee	Martin International Les Grands Explorateurs	National Institute of Allergy and Infectious Diseases	Agence Nationale de la Recherche	NSF	Swedish Research Councils VR and Formas	Japan Agency for Marine-Earth Science and Technology (JAMSTEC)
Reference paper	This paper	(6)	-	(57)	(58)	(59)	(33)	(7)	(13)	(34)	(36)	(37)	(36)
Published results elsewhere^a^	No	No	Yes	Yes	No	Yes	No	No	No	Yes	Yes	Yes	No
Exclusively marine	Yes	No	No	No	No	No	Yes	Yes	No	No	Yes	Yes	Yes
User registration for accessing data	Yes	Yes	No	Yes	No	No	No	No	No	No data deposition	Only upon request	No data deposition	No
Controlled user account creation	Yes	Yes	Yes	Yes	Yes	No	Yes	NA	Yes	No account creation	No account creation	No account creation	Yes
Geographic Browsing	Yes	Yes	Yes	No	Yes	No	No	Yes	No	No	Yes	No	Yes
Invertebrates	Yes	Yes	Yes	Yes	Yes	Yes	Yes	Yes	No^b^	Yes	Yes	Only Littorina saxatilis sequences	Yes
Vertebrates	Yes	Yes	Yes	Yes	Yes	Yes	Yes	Yes	No^b^	No	No	No	Yes
Microorganisms	Yes	No	Yes	Yes	Yes	Yes	No	No	Yes	Yes	Yes	No	Yes
Water quality	Yes	No	Yes	Yes	Yes	No	No	No	Yes^b^	No	No	No	No
Molecules	Yes	No	Yes	Yes	Yes	Yes	No	No	Yes	Yes	No	Yes	No
Spreadsheet	Yes	Yes	Yes	Yes	Yes	Yes	No	No	No	No	Yes	No	No
Video	Yes	No	Yes	Yes	Yes	No	No	No	No	No	No	No	Yes
Photo	Yes	No	Yes	Yes	Yes	No	Yes	No	No	No	No	No	Yes
Other	Any kind of file	Species occurrence (observations and checklists)	Yes	Compressed files	Any kind of file	Phylogenetic information files (e.g. nexus)	Species occurrence	Species distribution	FASTA files	FASTA files	FASTA files	FASTA files	Species occurrence
Analytical tools	No	No	No	No	No	No	No	Yes	Yes	Yes	No	Yes	No
Connectivity between databases	Yes	Yes	Yes	Yes	Yes	No	Yes	Yes	No	No	No	No	Yes

^a^If the database is marked as ‘Yes’, it means that the data published must be already published in a scientific or data paper.

### Relevance of BaMBa to the Brazilian EEZ exploration

Exploitation of marine resources generally advances faster than research on their management and conservation. Most of the time policy-makers are faced with limited data to support their decisions. Expanding and maintaining global databases on fauna and physical characteristics, including incorporating historical data and information about fisheries to support exploration impact evaluation was recommended (41). Recently, more attention has been paid to deep-sea mining impacts. Improved collaboration through information-sharing is suggested to overcome fragmented governance beyond international waters and seabed (42). Taking the example of Brazil, marine habitats are threatened by e.g. fishing, mining, eutrophication and coral disease (10, 43, 44). Abrolhos Bank rhodolith beds alone account for approximately 5% of the world’s total carbonate banks (20). Although Brazilian rhodolith beds have been the target of mineral exploration as a source of micronutrients and carbonates for agriculture (45, 46), the resilience, size and structure of the rhodolith beds have just recently been documented (18, 20). Developing countries ought to be prepared for the challenges ahead, but the preparedness is less clear concerning for their marine resources (47). Recent initiatives such as the Information System on Brazilian Biodiversity (SiBBr – http://www.sibbr.gov.br/), to which BaMBa is linked, offer an opportunity for improving governance of marine resources.

### Developing a strategy for BaMBa

Data integration is a challenging task in general (48). A strategy for BaMBa will comprise:

1. A broad testing and evaluation moment involving representative scientists and other stakeholders,
2. A further technical development: In the case of biodiversity and (meta)genomics, Robbins *et al*. (49) propose the evaluation of extensions required to promote interoperability between the two main standards in these fields, Darwin Core and MIxS, respectively. Some of these extensions are proposed in Tuama *et al.* (50). In the future, *late integration* techniques (51) might be used to facilitate this process. A tool called *Data Manager Library* (26) might also be used for this purpose since it can use metadata describing the columnar attributes of a dataset to extract its contents and store it in a relational database. The system supports integrating data from multiple datasets. It depends on the quality of metadata, i.e. how well it describes the logical schema of the dataset. In the long term, semantic web techniques have potential to provide robust solutions to data integration in biodiversity and (meta)genomics (52) by providing ontologies that describe the concepts and inference rules in each of the domains involved. BaMBa will follow these developments in order to further facilitate and improve data integration. From the data analysis point of view, it is important to expose the information stored in the integrated database in a way that is easy to consume that would enable better integration with statistical tools such as R (53), and scientific workflows management system (54), such as SciCumulus (55) and Swift (56). Tracking provenance in these scientific workflows (57) would be important in enabling reproducibility and validation of data analysis routines.
3. Involvement into specific scientific uses of BaMBa in the synthesis of data into a modeled ‘data landscape’ and into the (pre)modeling of marine systems.
4. Organize a meeting with the Brazilian stakeholders in marine biodiversity.

## Funding

Carlos Chagas Filho Research Foundation of the Rio de Janeiro State (FAPERJ); National Counsel of Technological and Scientific Development (CNPq); Coordination for the Improvement of Higher Education Personnel (CAPES). Funding for open access charge: 140869/2012-3 and 4848-14-9.

*Conflict of interest*. None declared.
